# *Pllans–II* Induces Cell Death in Cervical Cancer Squamous Epithelial Cells via Unfolded Protein Accumulation and Endoplasmic Reticulum Stress

**DOI:** 10.3390/molecules27196491

**Published:** 2022-10-01

**Authors:** Alejandro Montoya-Gómez, Nelson Rivera Franco, Leonel Ives Montealegre-Sanchez, Luis Manuel Solano-Redondo, Andrés Castillo, Mildrey Mosquera-Escudero, Eliécer Jiménez-Charris

**Affiliations:** 1 Grupo de Nutrición, Facultad de Salud, Universidad del Valle, Cali 760043, Colombia; 2 TAO-Lab, Centre for Bioinformatics and Photonics-CIBioFi, Department of Biology, Universidad del Valle, Cali 760032, Colombia; 3 Grupo Laboratorio de Herpetología, Facultad de Ciencias, Universidad del Valle, Cali 760043, Colombia

**Keywords:** snake venom, phospholipase A_2_, antitumor potential, transcriptomic analysis, bioprospecting

## Abstract

Due to the lack of chemotherapeutic drugs that selectively affect cervical cancer cells, natural sources such as snake venom are currently being investigated for molecules with antitumor potential. *Pllans–II*, a phospholipase A_2_ type–Asp49 from *Porthidium lansbergii lansbergii* snake venom, induced cell death in a cervical cancer cell line—Ca Ski—related to dysfunction in the ability to resolve endoplasmic reticulum stress, evidenced by sub–expression of genes such as PERK, ERO1 PDIs, HSP70, and CHOP. Western blot analysis validated the last two genes′ sub–expression at the protein level. In addition, *Pllans–II* presented a dose–dependent cytotoxic effect on cancer cells and an insignificant effect on healthy endothelial cells (HUVEC). Additionally, *Pllans–II* inhibited cancer cells′ adhesion and migration capacity, induced cell cycle arrest in the G2/M phase, and induced apoptosis stimulated possibly by the extrinsic route. These results demonstrate for the first time that *Pllans–II* has an antitumor effect on a squamous epithelial cervical cancer cell line and represents a possible biotechnological tool for designing a prominent antitumor agent.

## 1. Introduction

Cervical cancer (CC) is a malignant neoplasm originating in cervical cells. It is the fourth cause of morbidity and mortality for cancer in women and occupies ninth place in global types of cancer–causing mortality in both sexes. Approximately 604,000 cases of CC were reported throughout the world in 2020, and for the same year there were approximately 342,000 deaths due to this pathology [1].

Usually, CC develops in the cervical epithelium from cells infected with the human papillomavirus. It is mainly promoted by a suppression of apoptosis, caused by the expression of viral oncoproteins, which inactivate some apoptotic process effector proteins [2]. Platinum–based drugs, such as Cisplatin, are the most frequent treatment of the first line of chemotherapy against CC, and one of its target mechanisms is to reactivate the apoptotic process in cancer cells [3,4,5,6]. However, the lack of specificity of this type of treatment is an issue, as it affects cancer cells and also healthy cells. The chemotherapy significantly affects the quality of life of patients, leading in many cases to the abandonment of treatment, which contributes to the high death rate [7,8].

It is for this reason that, currently, one of the significant challenges for medicine is the exploration of new intervention strategies and the implementation of more effective drugs against CC which are less toxic for healthy tissues, which leads to further research for the finding of molecules that focus on the tumor microenvironment, particularly in the inhibition of metastasis and the induction of cell apoptosis [7]. Many molecules with such properties have been found in snake venoms, of which the outstanding examples are L–amino acid oxidases, disintegrins, type–C lectins, metalloproteinases, and phospholipases A_2_ [9,10,11,12]. The snake venom phospholipases A_2_ (svPLA_2_) are highly effective in affecting the development of tumor cells of different origins. However, the mechanisms by which they exert their effect have not been fully elucidated [13]. Some studies have demonstrated the ability of svPLA_2_ to interact through their C–terminal region with components of cell membranes, this being probably related to the selective effect that occurs on tumor cells [14,15,16,17]. The apparent svPLA_2_ mechanism is the induction of cell death due to an increase in intracellular oxidative stress, which is a mechanism widely recognized as a trigger for the activation of pathways that lead to cell cycle arrest and cell death [13,18,19]. These molecular processes have been evidenced in several cell lines treated with BthTX–I, a PLA_2_ isolated from the venom of *Bothrops jararacussu*, specifically affecting the G0/G1 phase [19]. The ability of cancer cells to proliferate and migrate has also been shown to be affected by treatment with BnSP–6, a PLA_2_ from the venom of *Bothrops pauloensis*, and this effect was related to DNA damage [20,21]. Other characteristics inherent to cancer that have been shown to be affected by svPLA_2_ (MVL–PLA_2_) are adhesion and migration, and this has been demonstrated in vitro and in vivo [22]. Recently, BthTX–II, a PLA_2_ from *Bothrops jararacussu* venom, demonstrated that the ability to induce cell cycle arrest, adhesion inhibition, migration, and cell death is related to modulation of the expression of genes related to extrinsic pathway apoptosis and with the modulation of integrin expression, both at the gene and protein levels. This alteration to integrins possibly generates interference for cancer cells to adhere to the extracellular matrix (ECM), which causes modifications in the cytoskeleton that lead cancer cells to decrease their metastatic potential and lead to death processes [23]. These data are consistent with the hypothesis that svPLA_2_ probably exert their antitumor effect by interaction with cell membrane receptors (such as cell growth factors receptors or integrins) that trigger signal transduction events, rather than by catalytic and cytotoxic activity of svPLA_2_ [24].

Our research group is studying a svPLA_2_ type Asp49 “*Pllans–II*” with cytotoxic activity on HeLa cells, a glandular epithelial cell line of CC that contain human papilloma virus 18 (HPV–18) [25,26,27]. However, the antitumor effect of *Pllans–II* has not yet been evaluated in any other cell line of cervical carcinoma. In this sense, Ca Ski, a human papillomavirus (HPV–16) associated with cervical cancer cell line, is relevant because it serves as a model for the study of advanced cervical carcinoma [28]. Given the evidence that supports the hypothesis that *Pllans–II* presents greater specificity on CC cells compared to non–cancer cell lines of mammals, the cytotoxic effect exerted by *Pllans–II* on the squamous epithelial of CC, Ca Ski, was evaluated for the first time, as well as the antimetastatic potential, the mechanism of induced cell death, and the alteration to the cell cycle. In addition, a transcriptomic analysis was carried out with a verification of some protein expression to obtain evidence of the cell death mechanism triggered.

## 2. Results

### 2.1. Pllans–II Induces Cytotoxicity in Ca Ski Cells without Damaging the Cytoplasmic Membrane

**Figure 1:**
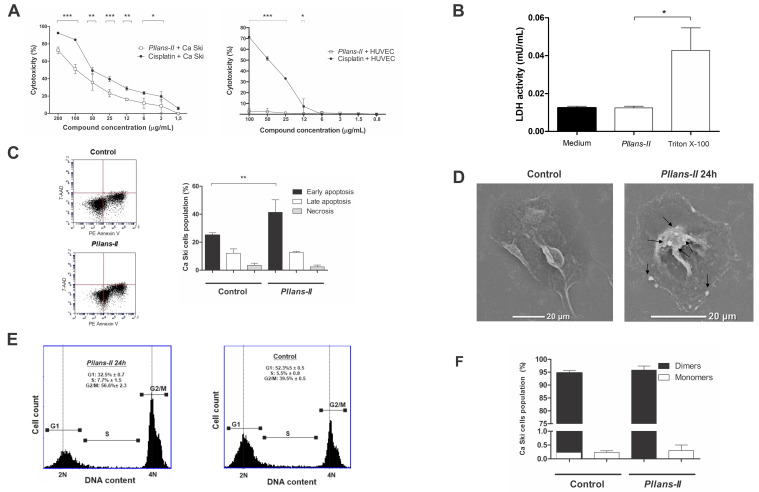
*Pllans–II* induces apoptotic death and cell cycle arrest in Ca Ski. (**A**) Cytotoxic activity of *Pllans–II* and Cisplatin^®^ on squamous epithelial cells of cervical cancer and non–tumorigenic cells; Ca Ski cells and non–tumorigenic line of human endothelial cells (HUVEC) were treated with concentrations of serial dilutions (200–1.5 μg/mL) of both *Pllans–II* and Cisplatin^®^. (**B**) Effects of *Pllans–II* (100 μg/mL) on lactate dehydrogenase (LDH) levels in Ca Ski cell medium after 24 h of treatment. Data are expressed as mean ± SD, and procedures were carried out in triplicate. (**C**) Flow cytometry analysis of cell death generated in Ca Ski cells by *Pllans–II* treatment (100 μg/mL). The dot plot revealed the percentage of cells in early apoptosis (lower right quadrant), late apoptosis (upper right quadrant), and necrosis (upper left quadrant). The bar chart shows the percentages of cells at each stage. (**D**) Morphological changes induced by *Pllans–II* (100 μg/mL) on Ca Ski cells, detected by scanning electron microscopy (SEM). The formation of apoptotic bodies (marked with arrows) and size reduction is evident on *Pllans–II* treated Ca Ski cells. (**E**) Effect of *Pllans–II* (100 μg/mL) on the cell cycle of Ca Ski cells. The DNA content in each phase of the cell cycle was analyzed by flow cytometry. *Pllans–II* induced a significant decrease in the number of 2N cells in G1 phase and an increase in the number of cells in G2/M phase compared to the control after 24 h of incubation. (**F**) Effect of *Pllans–II* on the mitochondrial membrane potential in Ca Ski cells. The alteration to Δψm was analyzed by flow cytometry staining with JC–1, after the cells were incubated with 100 µg/mL of *Pllans–II* and RPMI 1640 medium as a control. At the time of evaluation, the percentages of cells found with the JC–1 probe in monomeric and dimeric form for each treatment are shown. There are no statistically significant differences between treatments. Statistically significant differences are observed with *** *p* < 0.001, ** *p* < 0.01, and * *p* < 0.05.

The PLA_2_ *Pllans–II*, previously purified, identified, and characterized by Jimenez–Charris et al. [26], displayed a cytotoxic effect in a dose–response manner, up to approximately 75% cell death at 200 µg/mL. Furthermore, cell viability was reduced to 49% with 100 μg/mL of *Pllans–II*, considering this concentration as the IC50 of the protein. On the other hand, no cytotoxic effect on HUVEC cells at different concentrations of *Pllans–II* was detected (Figure 1A).

The cytotoxic effect of *Pllans–II* was compared with that exerted by Cisplatin, a chemotherapeutic drug used in the treatment of CC. Cisplatin had a significantly higher cytotoxic effect on Ca Ski cells; however, its toxic effect on non–cancer human cells was more significant than *Pllans–II*, which was almost innocuous (Figure 1A). In addition, no difference in LDH activity was detected in the culture medium of cells treated with *Pllans–II* compared with the controls, indicating that the protein does not alter the integrity of the cytoplasmic membrane. (Figure 1B).

### 2.2. Pllans–II Induces Apoptosis and Cell Cycle Arrest in the G2/M Phase

The proportion of cells in early apoptosis (annexin–V positive and PI negative) was significantly higher for treatment with *Pllans–II* than for control after 24 h of incubation (41.38% vs. 25.01%, respectively. *p* < 0.01, *n* = 3). No statistically significant differences were observed between the treatments when late apoptosis and necrosis were evaluated (Figure 1C).

In cells treated with *Pllans–II*, apoptotic bodies were observed in the cytoplasmic membrane, with a reduction in cell size compared to the control cells (Figure 1D).

In addition, flow cytometry analysis revealed that *Pllans–II* affected the cell cycle, with more cells in the G2/M phase (56.6%) and fewer cells in the G1 phase (32.5 %) compared to the control treatment. The percentage of cells in the S–phase was not significantly affected between treatments (Figure 1E).

### 2.3. Pllans–II Did Not Generate Disruption in Mitochondrial Membrane Potential on Ca Ski Cells

The Δψm was analyzed by flow cytometry after staining the cells with the JC–1 dye (Figure 1F). The analyses revealed no significant difference in the proportion of the dye in its monomeric and dimeric form inside the cells between the control and the treatment. Most cells had the dye in its dimeric form, which indicates that mitochondrial membranes did not lose permeability when treated with *Pllans–II*.

### 2.4. Pllans–II Induces Changes in Ca Ski Cells Gene Expression

**Figure 2:**
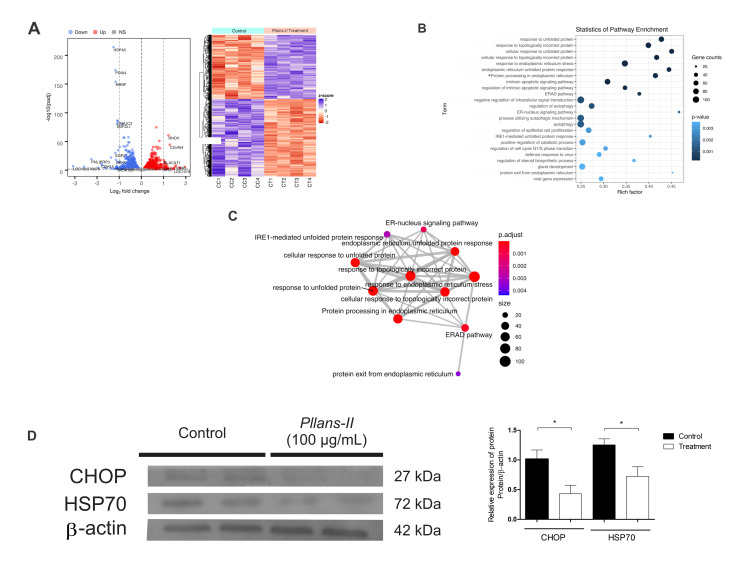
*Pllans–II* does not affect mitochondrial integrity but alters genes’ expression related to the response to endoplasmic reticulum stress. (**A**) Analysis of differentially expressed genes (DEG) from Ca Ski cells treated with *Pllans–II* and control. The red dots in the volcano plot represent genes with significantly higher expression in the treatment (up–regulated), while the blue dots represent genes significantly sub–expressed in the treatment group (down–regulated). The gray dots indicate genes that are not differentially expressed. The heat map shows changes in gene expression for each of the treatment and control samples. The red and blue colors represent, respectively, the increase and decrease in expression, while the white color indicates no change. (**B**) Pathway enrichment analysis. The term (Y–axis) represents the identification code and the pathway for the GO and KEGG databases. Asterisk (*) indicates the pathway found significant for KEGG. The enrichment factor (X–axis) represents the relationship between the number of differentially expressed genes and the total number of genes in a given pathway. The area of each colored circle is proportional to the number of genes involved in each pathway; the color indicates the *p*–adjusted value. (**C**) The most affected functional module of enriched terms map from Ca Ski cells treated with *Pllans–II*. The relation of the top–30 significantly richer terms (by *p*–adjusted values) are indicated by the thickness of the lines and represent the amount of DEG shared between each pair of terms. The size of each node indicates the gene count (number of genes enriched in each category) and the color represents the adjusted *p*–value. (**D**) Western blot analysis of HSP70, CHOP, and Actin protein after treatment with *Pllans–II* (100 μg/mL). The densitometry of bands was quantified by ImageJ software and represented in a histogram.

The gene expression evaluated between the control and treatment groups is reported through a hierarchical grouping (heatmap analysis) and a volcano plot (Figure 2A). A total of 2694 DEG were identified; these included 1222 and 1472 down–regulated and up–regulated genes, respectively. PCA showed that the gene expressions of the treated cells are well differentiated from the controls (Figure S1). In addition, 97 of these DEG presented fold–change values greater than 1 or less than −1, among them HSPA5, PDIA4, MANF, PERK, HSP70, and ERO1, which are involved in protein folding and ER function.

### 2.5. Pllans–II Affects Expression of Genes and Proteins Related to Response to Misfolded Proteins and Apoptosis

A total of 192 significantly enriched terms were found for GO analysis within the category of biological processes (BP) and a single significantly enriched term for KEGG pathway analysis. Figure 2B reports the 23 most enriched GO biological process and the significantly enriched pathway of KEGG (Figure S2). It is observed that the enrichment factor for these terms is between 0.25 and 0.47. When organizing these terms by their significance, two groups can be differentiated: (1) In the first group are the two most significant terms, which are related to proteins misfolding in the endoplasmic reticulum (ER); (2) In the second group, there are terms related to intrinsic apoptotic pathways signaling. The remaining processes are related to highly varied pathways, such as autophagy, regulation of epithelial cell proliferation, defense response to virus, and regulation of steroids biosynthetic process, among others.

The enrichment map can differentiate four main functional modules, three smaller clusters with only two pathways each, and two orphan pathways (data not shown). The first module was the largest and most significant, mainly related to the response of incorrectly folded proteins and protein processing in the ER (Figure 2C). The other three main modules were related to apoptosis, autophagy, and protein localization in ER.

Since protein misfolding and ER processing were altered in the gene expression analysis, a Western blot analysis was carried out to evaluate the expression of the proteins HSP70 (involved in protein folding and the response to misfolded proteins) and CHOP (involved in the stress response in ER and apoptosis); a decrease in the concentration of both proteins in Ca Ski cells was found when treated with *Pllans–II* (Figure 2D).

### 2.6. Pllans–II Effects on Ca Ski Cells Adhesion and Migration

**Figure 3:**
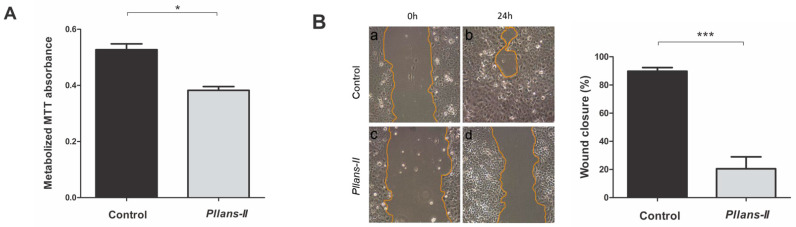
*Pllans–II* affects Ca Ski cells adhesion and migration. (**A**) Effect of *Pllans–II* on Ca Ski cell adhesion. Cells were incubated in RPMI medium alone (control) or with 100 μg/mL *Pllans–II* for 24 h. Metabolic activity was determined by MTT assay. (**B**) Migration effect of *Pllans–II* on Ca Ski cells by the wound healing assay. Cells were seeded at 5 × 10^4^ cells/well in 24 well plates until obtaining complete confluence. After 24 h, the medium was discarded, and the confluent monolayer was scratched with a 10 μL pipette tip to create an area devoid of cells (0 h) (**a**,**c**). After this process, cells were treated in the (**a**) absence or (**c**) presence of *Pllans–II* at 100 µg/mL. Pictures were taken at the beginning 0 h, (**a**,**c**) and the end of the experiment 24 h, (**b**,**d**), using an inverted optical microscope (Nikon Eclipse TS100). The bar graph shows the percentage of cell migration for *Pllans–II* and control after 24 h of incubation. The data are expressed as means ± SD (*n* = 3). Statistically significant differences are observed between treatments with * *p* < 0.05 and *** *p* < 0.001.

The ability of *Pllans–II* to inhibit cell adhesion and migration was evaluated by the MTT colorimetric assay and the wound healing assay, respectively. As shown in Figure 3A, the percentage of cells that metabolized MTT was 20% higher in the control indicating a significant inhibition of cell adhesion for treatment with the protein. Additionally, the migratory capacity of the cells treated with *Pllans–II* was highly inhibited after 24 h compared to control (Figure 3B).

## 3. Discussion

Several studies have reported that svPLA_2_s represent a potential use in designing anticancer drugs [29,30,31,32]. *Pllans–II* developed a cytotoxic effect on Ca Ski cells, and unlike Cisplatin, the svPLA_2_ did not show significant cytotoxic effects on normal endothelial cells. This specificity of svPLA_2_s for tumor cells is not necessarily related to enzyme activity, and interactions involving non–catalytic regions of the protein with membrane receptors may be implicated [24], which is consistent with the finding that *Pllans–II* treatment did not alter the cytoplasmic membrane integrity. Furthermore, such interactions could affect intracellular pathways related to tumor cells viability, such as increases in apoptosis [24,33]. In the present work, evidence of cell death activation by apoptosis was demonstrated as one of the mechanisms involved in the effect of *Pllans–II* over Ca Ski cells. In addition, the cell membrane modifications that were detected in the study (phosphatidylserine phospholipids translocated to the extracellular region) help recognition and clearance by the immune system’s phagocytes [34]. The morphological modifications observed in Ca Ski cells support the hypothesis that *Pllans–II* induces apoptosis, since these had been described as visual marks of the apoptosis process [35].

The arrest of the cell cycle in cancer cells is a desired property in a chemotherapeutic drug candidate [36]. In this regard, the analysis by flow cytometry confirmed that *Pllans–II* generated an arrest in the G2/M phase of the cell cycle. Furthermore, such arrest may be related to apoptosis, since both processes have in common the activation of a set of regulatory proteins, such as Chk1, Chk2, CDC2, CDC25, and CDNK1, which have been found to be altered after a cell cycle arrest in this phase in other investigations [36,37,38].

Studies with toxins from snake venoms on cancer cells indicate that the primary mechanisms by which pro–apoptotic activity is exerted are activation of BCL–2 family proteins, disruption of mitochondrial membrane potential, and subsequent release of cytochrome C (intrinsic pathway) [7,39]. However, Jiménez–Charris et al. [27] found that HeLa cells, under the action of *Pllans–II*, overexpress genes involved in the extrinsic apoptotic pathway with a down–regulation of some genes of the intrinsic pathway. These results are similar to the findings of the present study, where Δψm was not affected in Ca Ski cells, which strengthens the hypothesis that *Pllans–II* generates cell death without significantly affecting mitochondrial integrity or the activation of the intrinsic apoptotic pathway since genes of the BCL–2 family were over expressed and genes that encode for proteins that participate in mitochondrial permeabilization, such as CHOP and Bak/Bax, were sub–expressed.

The apoptotic Ca Ski cell death should be related to alterations in the gene expression involved in the ER stress response associated with protein misfolding. In this study, the most altered genes encode for proteins that participate in the protein folding in ER and ER stress. For example, HSPA5 is a crucial component of the unfolded protein response (UPR) since it promotes cell survival under conditions of ER stress [40]; PDIA4 corresponds to an isoform of the protein disulfide isomerase (PDI), which participates in the oxidative folding of proteins (OPF) that occurs in the ER [41]; mesencephalic astrocyte–derived neurotrophic factor (MANF) is also a protein that increases its expression when there are endoplasmic reticulum stress events [42]; and PERK, which contributes to the inhibition of the synthesis of proteins that otherwise continue to accumulate in the ER lumen [43]. These observations suggest that *Pllans–II* generates down–regulation of these genes’ expression, affecting Ca Ski cells’ ability to resolve a misfolding protein environment and increasing the stress to ER, contributing to the Ca Ski cells’ death.

Additionally, our analysis of altered biological pathways revealed that several genes associated with the ER–associated degradation (ERAD) pathway were altered, such as Ubc 6/7, ERO1, Derlin, HSP70, and Hsp40. These genes encode for proteins that can ubiquitinate and degrade misfolded proteins accumulated in the ER [44], and treatment with *Pllans–II* may induce a failure in the endoplasmic reticulum stress response that ultimately leads to cancer cell death. The Western blot evaluation helped us to confirm a decrease in the concentration of one of these proteins, HSP70, as was shown in the gene expression analysis.

Recent research has shown that the accumulation of dysfunctional proteins can exacerbate ER stress due to ROS imbalance that disrupts ER redox homeostasis [43]. Our results suggest that the response to ER protein misfolding is altered in Ca Ski cells treated with *Pllans–II*, which may be related to alteration of redox homeostasis since the flavoprotein ERO1 was found to be downregulated, which has an essential activity in OPF by acting on PDIs. By decreasing the PDI activity, the ability to perform OPF is lowered, and this, in addition to increasing the stress to ER, generates an imbalance in ROS [45,46].

Previous works with *Pllans–II* suggested that cytotoxic activity may be associated with interaction with integrins α5β1, a dimeric ECM adhesion protein that modulates survival, proliferation, angiogenesis, and protection pathways against apoptosis [47,48,49]. As this integrin is overexpressed in CC cells [50] and our results on inhibition of cell adhesion, as well as on proliferation and migratory capacity, are congruent with the findings of Jimenez–Charris et al. (2019) [27], we propose that interactions of *Pllans–II* with α5β1 could be occurring with Ca Ski cells. Interestingly, inhibition of the interaction of integrins with ECM may also be related to a type of apoptosis known as *anoikis,* a type of cell death promoted by lack of anchorage to the ECM. This lack of anchorage causes modifications in the cytoskeleton, and survival signals given by binding to the ECM are no longer transduced. Apoptosis regulatory kinases are involved in this type of death, such as focal adhesion kinase (FAK), integrin–linked kinase (ILK), and Src kinases, proteins that are active when integrins bind to the ECM [51,52]. *Anoikis* is also related to ER stress, evidenced by transcriptomic analysis in this work. Several studies have reported that activation of the PERK axis of the UPR to relieve reticulum stress promotes tumor cell survival by inhibiting oxidative stress and *anoikis* [53,54,55,56]. Specifically, PERK and some genes that code for proteins downstream of PERK, such as CHOP and GADD34, were found to be sub–expressed in Ca Ski after treatment with *Pllans–II*, and the CHOP protein was shown to be sub–expressed in Western blot. Additionally, the modifications to the cytoskeleton in *anoikis* may also be related to the over–expression evidenced for RHOV. This gene codes for Rho GTPases, proteins responsible for maintaining cytoskeletal dynamics regulated for the cell adhesion process [57].

**Figure 4:**
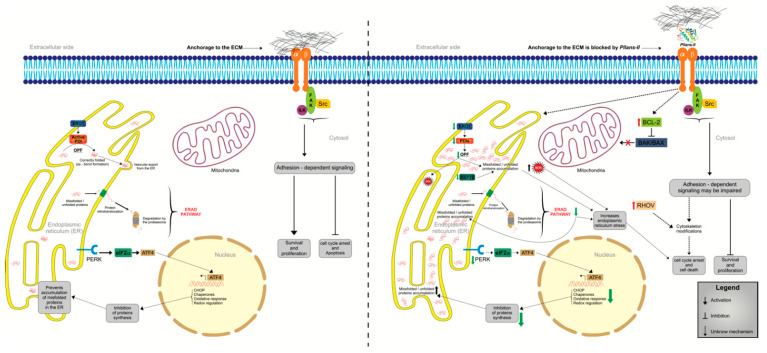
Representative model of antitumoral effects of *Pllans–II* on Ca Ski cells. The left part represents normal processes in untreated Ca Ski cells, where the ECM anchoring processes, the unfolded protein response (UPR), and the ER–associated protein degradation system (ERAD), develop in the endoplasmic reticulum, keeping the cells viable. The right part represents the possible effect of *Pllans–II* on cervical tumor cells. *Pllans–II* interferes initially with the binding of integrins to the ECM, which would affect cell adhesion and migration. In addition, this interaction could interfere with signaling pathways in the cell and modify the expression profile of genes related to UPR, mitochondrial permeabilization, and the ERAD pathway, which is usually responsible for the clearance of misfolded/unfolded proteins in ER. These effects would generate an increase in the stress on the endoplasmic reticulum in treated Ca Ski cells and not resolve it, leading to arrest in the cell cycle and apoptosis. Red arrows mean up–regulation, and green arrows mean down–regulation.

To summarize the results obtained, Figure 4 represents the proposed model of action of *Pllans–II* on Ca Ski cells. The first event would be the interaction of PLA_2_ with extracellular matrix–binding transmembrane receptors. We suggest that *Pllans–II* would prevent the cells from binding to ECM proteins, thereby ceasing to trigger intracellular signaling events that typically allow cancer cells to maintain their immortal phenotype. In addition, the interaction of *Pllans–II* with Ca Ski receptors would also modulate the expression of genes that encode for proteins essential in the resolution of ER stress and proteins involved in the induction of cell death by apoptosis.

As a consequence of the results obtained in the present work, our group is currently working to elucidate the complete amino acid sequence of the protein. With these results, it will be possible to perform in silico experiments, such as molecular docking, to confirm pharmacological receptors with which *Pllans–II* interacts to induce the observed effects. In addition, obtaining the complete sequence will allow us to develop in vitro molecular labeling experiments to demonstrate the cellular compartment in which the protein interacts to generate the observed cytotoxic effect.

## 4. Materials and Methods

### 4.1. Venom Collection and Pllans–II Purification

The snake venom from *Porthidium lansbergii lansbergii* was obtained from specimens collected in the Atlantic department, Colombia. Snakes were released after venom collection, and all samples were pooled, centrifuged to remove debris, dried in a vacuum centrifuge, and stored at −20 °C until their use. Sample collection was allowed by Autoridad Nacional de Licencias Ambientales–ANLA, Resolution number 1070, 28 August 2015, and a contract for access to genetic resources and their derivative products No. 307, 3 March 2021, was granted by Ministerio de Ambiente of Colombia to develop the present investigation.

Venom pools were resuspended in H_2_O/0.1% trifluoroacetic acid (TFA), *Pllans–II* protein was obtained using RP–HPLC in the same manner described in (27). In brief, aliquots of venom (2 mg) were separated in a Zorbax SB–C18 column (250 × 4.6 mm, 5 μm particle diameter; Agilent) eluted at 1 mL/min with a gradient from H_2_O/0.1% TFA to acetonitrile/0.1% TFA. The eluent was monitored at 215 nm in an Agilent 1260 Infinity chromatograph using the EZ CHROM ELITE software, version 4.9.005.0 (Agilent Technologies, Inc. 2006, Santa Clara, CA, USA). The target svPLA_2_ fraction was collected manually, dried by vacuum centrifugation, and stored at −20 °C.

### 4.2. Cell Culture and Cytotoxicity Assay

Ca Ski and HUVEC cell lines were obtained from the American Type Culture Collection (ATCC; Manassas, VA, USA) and maintained at 37 °C in a humidified incubator containing 5% CO_2_ atmosphere. RPMI 1640 medium containing 10% FBS, 2 mM L–glutamine, 2 mM sodium pyruvate, 1 mM non–essential amino acids, 100 U/mL penicillin, and 100 mg/mL streptomycin were used to culture the cell lines. When confluency was reached, the cells were divided for the experiments.

The cytotoxicity activity of *Pllans–II* was evaluated on Ca Ski and HUVEC cell cultures by the colorimetric MTT assay. Briefly, the cells (4 × 10^4^ cells/well) were seeded into a 96–well microplate. Adhering cells were incubated 24 h with the complete medium in the absence (control) or presence of serial dilution concentrations of *Pllans–II* (200, 100, 50, 25, 12.5, 6.3, 3.1, 1.6, and 0.8 μg/mL) for 24 h at 37 °C and 5% CO_2_. After treatment, the cells were incubated with MTT (5 mg/mL, 20 μL/well 3-(4,5-dimethylthiazol-2-yl)-2,5-diphenyl tetrazolium bromide) for 3 h at 37 °C, and the formazan crystals were dissolved by 100 μL of 10% SDS and 0.01 M HCl followed by incubation for 18 h at 37 °C and 5% CO_2_. The absorbance was read after 18 h on a multi–well scanning spectrophotometer at 570 nm in a microplate reader (iMark ^®^ Microplate Absorbance Reader–BioRad, Hercules, CA, USA). The cytotoxic activity of our experimental protein was compared with Cisplatin^®^ to determine the effectiveness of *Pllans–II* as a possible anti–tumor therapeutic agent.

### 4.3. Inhibition of Adhesion and Wound Healing Assay

The potential of *Pllans–II* to inhibit adhesion on Ca Ski cells was quantified by the MTT colorimetric assay, as previously described [58]. Briefly, the cells (3 × 10^4^ cells/well) were pre–incubated in the absence (control) or presence of *Pllans–II* (IC_50_ = 100 μg/mL, determined in the cytotoxicity test) for 30 min at 37 °C. Unattached cells were removed by washing three times with HBSS, whereas the viability of attached cells was measured by incubating with MTT for 3 h at 37 °C. Then, 100 μL/well of 10% SDS and 0.01 M HCl was added and incubated for 18 h at 37 °C and 5% CO_2_. The absorbance was read at 570 nm in a microplate reader (iMark ^®^ Microplate Absorbance Reader–BioRad, Hercules, CA, USA). The wound healing assay was measured according to with some modifications [27,58].

Tumorigenic migration inhibition was tested by the wound healing assay [59] with some modifications. Briefly, cells were seeded at 5 × 10^4^ cells/well in 24 well plates. After 24 h, the confluent monolayer was scratched with a 10 μL pipette tip to create an area devoid of cells, and the medium was discarded. After this process, the new medium alone (control) or containing *Pllans–II* IC_50_ (100 μg/mL). Cellular growth was evaluated after 24 h. Confluency was determined using an inverted microscope (Nikon Eclipse TS100, Shinagawa-Ku, Tokio, Japan), percentage of closure was quantified using the ImageJ software.

### 4.4. Lactate Dehydrogenase (LDH) Assay

To detect whether the cell membrane was damaged by treatment, LDH release into the culture medium was measured. Briefly, Ca Ski cells (2 × 10^4^/well) were seeded in 96 well plates and incubated with *Pllans–II* (100 μg/mL) or culture medium (control) or Triton X–100 (positive control) at 37 °C with 5% CO_2_ for 24 h. After incubation, 50 μL of the medium was collected, and LDH activity was quantified using the LDH Cytotoxicity Assay Kit following the manufacturer protocol (Sigma–Aldrich Catalog Number MAK066, Billerica, MA, USA), product formation was measured on a microplate reader (iMark ^®^ Microplate Absorbance Reader–BioRad, Hercules, CA, USA) at 450 nm.

### 4.5. Apoptosis and Cell Cycle Analysis

Ca Ski cells (5 × 10^5^/well) were placed in 12–well culture plates, maintained at 37 °C in 5% CO_2_ for 24 h, and incubated with a medium in the absence (control) or presence of *Pllans–II* (100 μg/mL). The apoptosis was measured using Apoptosis Detection Kit (BD Biosciences, Catalog number 559763, San Diego, CA 92121, USA) following the manufacturer’s protocol. Briefly, cells were resuspended in 1X binding buffer and 5 μL of Annexin V–PE and 5 μL of 7–AAD were added for subsequent staining in the dark for 15 min. Finally, apoptosis analysis was performed by flow cytometry (BD Accuri ™ C6–Biosciences, San José, CA 95131, USA) and the percentages of apoptotic and necrotic cells were determined using BD Accuri C6 software, version 1.0.264.15 (San José, CA 95131, USA).

The analysis of cell cycle arrest induced by *Pllans–II* on Ca Ski cells was analyzed by flow cytometry, as Krishan described previously [60] and following the modification made by [27]. Briefly, the Ca Ski cells (5 × 10^5^ cells/well) were treated with 100 μg/mL of *Pllans–II* or culture medium (control) for 24 h at 37 °C and 5% CO_2_. Then, cells were collected and fixed in 70% ethanol overnight at 4 °C. For each group, cells were incubated with propidium iodide (PI) (10 μg/mL) and RNase A (100 μg/mL) for 45 min at 37 °C in the dark. The cell cycle was analyzed by quantifying the DNA content using flow cytometry (BD Accuri ™ C6–Biosciences, San José, CA, USA), and data were analyzed using BD Accuri C6 software, version 1.0.264.15 (San José, CA, USA).

### 4.6. Visualization of Morphological Alterations Induced by Pllans−II

The morphological alterations in Ca Ski cells after exposure to *Pllans–II* were observed by scanning electron microscopy (SEM), as described by [61], with slight modifications. Briefly, cells (7 × 10^5^/well) were seeded in circular glass plates, immersed in culture plate wells, and kept at 37 °C with 5% CO_2_ for 24 h. After this, the cells were incubated for 24 h with medium in the absence (control) or presence of *Pllans–II* (100 μg/mL). Then, the cells were fixed with glutaraldehyde, formaldehyde, and phosphate buffer and dehydrated with different ethanol concentrations (20, 40, 60, 80, 90, and 100%). Finally, the samples were dried at room temperature, bathed with a thin layer of plasma–induced gold, and analyzed in a NeoScope JCM–5000 (NIKON/JEOL, Tokyo, Japan).

### 4.7. Mitochondrial Membrane Potential Analysis

The change in mitochondrial membrane potential (Δψm) on Ca Ski cells was determined using the Mitochondrial Membrane Potential Detection Kit (Agilent Technologies, La Jolla, CA, USA), following the manufacturer’s protocol. Briefly, cells (5 × 10^5^/well) were seeded in 12 well culture plates and kept at 37 °C with 5% CO_2_ for 24 h. After this time, the cells were incubated with medium either in the absence (control) or in the presence of *Pllans–II* (100 µg/mL) for 24 h. Subsequently, the cells were resuspended in 1X solution with the JC–1 probe and incubated at 37 °C with 5% CO_2_ for 15 min, to later be washed and centrifuged. Finally, the pellet was resuspended in 1× assay buffer, and the percentage of cells with the probe in aggregated/monomeric form was determined by flow cytometry (BD Accuri ™ C6–Biosciences, San José, CA, USA).

### 4.8. Transcriptomic Analysis

The cells (7 × 10^5^/well) were seeded in 12 wells plates and kept at 37 °C with 5% CO_2_ for 24 h. After this time, the cells were incubated for 24 h with medium, in the absence (control) or presence of *Pllans–II* (100 μg/mL). Total RNA was extracted using the RNeasy Micro kit (Cat. No. 74004, Valencia, CA, USA). The purity, integrity, and potential contamination of the extracted RNA was verified in a Nanodrop 2000 (Thermo Scientific, Waltham, MA, USA) (OD 260/280), in a bioanalyzer Agilent 2100 (Agilent, Santa Clara, CA, USA), as well as in an agarose gel.

The selected samples had no genomic DNA or protein contamination and a RIN number equal or major than 9. The NEB library construction was developed. Briefly, the mRNA was purified from total RNA using poly–T oligo–attached magnetic beads. The mRNA was fragmented randomly by adding a fragmentation buffer. Then, first–strand cDNA was synthesized using random hexamer primer and M–MuLV Reverse Transcriptase (RNase H); second–strand cDNA synthesis was subsequently performed using DNA Polymerase I and RNase H. Double–stranded cDNA was purified using AMPure XP beads. The remaining overhangs of the purified double–stranded cDNA were converted into blunt ends via exonuclease/polymerase activities. After adenylation of 3′ ends of DNA fragments, NEBNext Adaptor with hairpin loop structure was ligated to prepare for hybridization. The library fragments were purified with the AMPure XP system (Beckman Coulter, Beverly Hills, CA, USA) to select cDNA fragments preferentially 150–200 bp in length. The final library was obtained by PCR amplification and purification of PCR products by AMPure XP beads. Q–PCR was used to accurately quantify the effective library concentration (>2 nM) to ensure library quality. Finally, libraries were fed into Illumina machines for sequencing.

For the mapping of readings and quantification of expressions, the quality of the raw reads was evaluated with the FastQC software, version 0.11.9, Babraham Institute bioinformatics Group’s, Cambridge, UK (Andrews 2010), and the data were cleaned by eliminating adapter sequences, PCR duplication, empty and/or with low scores quality using the FASTX–ToolKit software, version 0.0.13, Hannon Lab, New York, USA (RRID: SCR_005534). The clean reads were mapped to the GRCh38.p12 human reference genome using STAR software, version 2.7.0f, Cold Spring Harbor Laboratory, New York, USA [62]. The expression levels of the genes were determined as raw counts with the featureCounts program of the subRead package [63]. For the analysis and annotation of differentially expressed genes (DEG), exploratory principal components analysis (PCA) graphs were made, and the distribution and density of gene expression were compared between each sample (Figure S1). These quality controls suggested that the data qualified for further analysis. The normalization of the counts and the differential expression analysis were performed using R’s DESeq2 package [64]. The Benjamini–Hochberg method was used to adjust the *p* values, which controls the false discovery ratio (FDR). DEG were identified if the *p*–adjusted value was <0.01. The volcano plots were created with R’s Enhanced Volcano package [65] to visualize differential gene expression. Likewise, a heat map was used to represent the expression of the DEG for each of the individuals within the treatment and control groups.

For the DEG pathway and functional enrichment analysis, the R cluster Profiler package [66] was used to identify the biological implications of DEG with the Gen Ontology database (GO, http://geneontology.org, Accessed date: 11 May 2022) within the category of biological processes (BP). In addition, a pathway analysis was performed with the Kyoto Encyclopedia of Genes and Genomes (KEGG, https://www.genome.jp/kegg/, Accessed date: 11 May 2022) database to identify biologically relevant pathways associated with DEG. Fisher’s exact test was used to select significant pathways based on adjusted *p* values, and those with a value < 0.05 were considered significant. From the 30 most significant enriched terms, an enrichment map was obtained with the enrichplot package of R [67], in which the sets of DEG shared between each pair of terms are related by lines, which facilitates the identification of functional modules.

### 4.9. Western Blot Analysis

Ca Ski cells were seeded in 6 well plates (10^6^ cells/well) in duplicate and incubated overnight. Next, cells were treated with *Pllans–II* (100 μg/mL) or culture medium (control) for 24 h. After treatment, cells were harvested in lysis buffer (C3228, CelLytic™ MT Cell Lysis Reagent, Sigma–Aldrich, St Louis, MO, USA) with protease inhibitor cocktail (04693116001, cOmplete™ Protease Inhibitor Cocktail, Roche, Mannheim, Germany), incubated for 15 min on a shaker, and centrifuged at 20,000× *g* for 10 min at 4 °C. The amount of protein was verified by the bicinchoninic acid (BCA) method, and 20 μg of each protein extract was mixed with load buffer in reduced conditions and loaded onto reducing 12.5% SDS–PAGE gels for proteins separation. The separated proteins were electrophoretically transferred onto nitrocellulose membranes (Whatman Protran BA85, Piscataway, NJ, USA), and membranes were blocked in TBST buffer (20 mM Tris, 137 mM NaCl, and 0.1% Tween–20, pH: 7.6) containing 5% low–fat milk. Then, membranes were incubated overnight with antibodies (1:1000) against CHOP (PA5–88116, ThermoFisher Scientific, Waltham, MA, USA) and HSP70 (PA5–28003, ThermoFisher Scientific, Waltham, MA, USA). Beta Actin monoclonal antibody was used as housekeeping (MA5–15739, ThermoFisher Scientific, Waltham, MA, USA). Next, the membranes were washed two times in TBST and incubated for 1 h with a secondary antibody with 1:10,000 dilution (anti–mouse IgG–peroxidase antibody produced in rabbit (A9044, Sigma–Aldrich, St Louis, MO, USA) for anti–actin, and anti–rabbit IgG–peroxidase antibody produced in goat (A0545, Sigma–Aldrich, St Louis, MO, USA) for anti–CHOP and anti–HSP70). The immunoreactivity signals were visualized by autoradiography using the chemiluminescent kit SuperSignal™ West Pico PLUS Chemiluminescent Substrate (34577, ThermoFisher Scientific, Carlsbad, CA 92008, USA). Densitometry of protein bands was quantified using Image–J software, version 1.53c (NIH, USA) and normalized to β–actin.

### 4.10. Statistical Analysis

All experiments were performed in triplicate. The results were expressed as mean ± SD. The difference significance between the means of treatment and control was determined by Student’s *t*–test or one–way ANOVA, whereas the comparison of two or more variables was assessed by two–way ANOVA, followed by the Bonferroni post–test using the software GraphPad Prism version 5 (GraphPad Software, Inc., San Diego, CA, USA), where *p* < 0.05 was considered significant.

## 5. Conclusions

This research showed that *Pllans–II* has a cytotoxic effect on Ca Ski cells, a squamous cell carcinoma of the cervix, by induction of apoptosis, cell cycle arrest in the G2/M phase, and failure in the ER stress response. This cytotoxic capacity seems to be related to the junction blockage between cancer cells and the extracellular matrix, which also reduces the ability to adhere and migrate, and, therefore, reduces the metastatic potential of this type of cells. In addition, no evidence of mitochondrial membrane alterations was observed, suggesting the activation of cell death via an extrinsic mechanism. The results obtained in this study through transcriptomic analysis and Western blot suggest that *Pllans–II* affects Ca Ski cells’ ability to resolve ER stress, which may be related to *anoikis*, a type of cell death promoted by the de–anchoring of cells to the ECM. For future work, it would be interesting to confirm, by differential proteomics, the intracellular pathways that may be affected in CC cells by *Pllans–II*, allowing us to know the action of the protein’s mechanism.

## Data Availability

The data presented in this study are available in the article and the supporting information.

## Supplementary Materials

The following supporting information can be downloaded at: https://www.mdpi.com/article/10.3390/molecules27196491/s1, Figure S1: Quality control for transcriptomic analysis. Figure S2: Enriched pathway of KEGG (hsa04141) with FC for each gene.
