# Supplementary material for: Pllans–II Induces Cell Death in Cervical Cancer Squamous Epithelial Cells via Unfolded Protein Accumulation and Endoplasmic Reticulum Stress
Source: Molecules. 2022 Oct 1;27(19):6491. doi: 10.3390/molecules27196491 (PMC9573087; doi:10.3390/molecules27196491)
Supplement: Supplementary file 1 [file molecules-27-06491-s001.zip › molecules-1934165-supplementary.pdf]

## Supplementary material

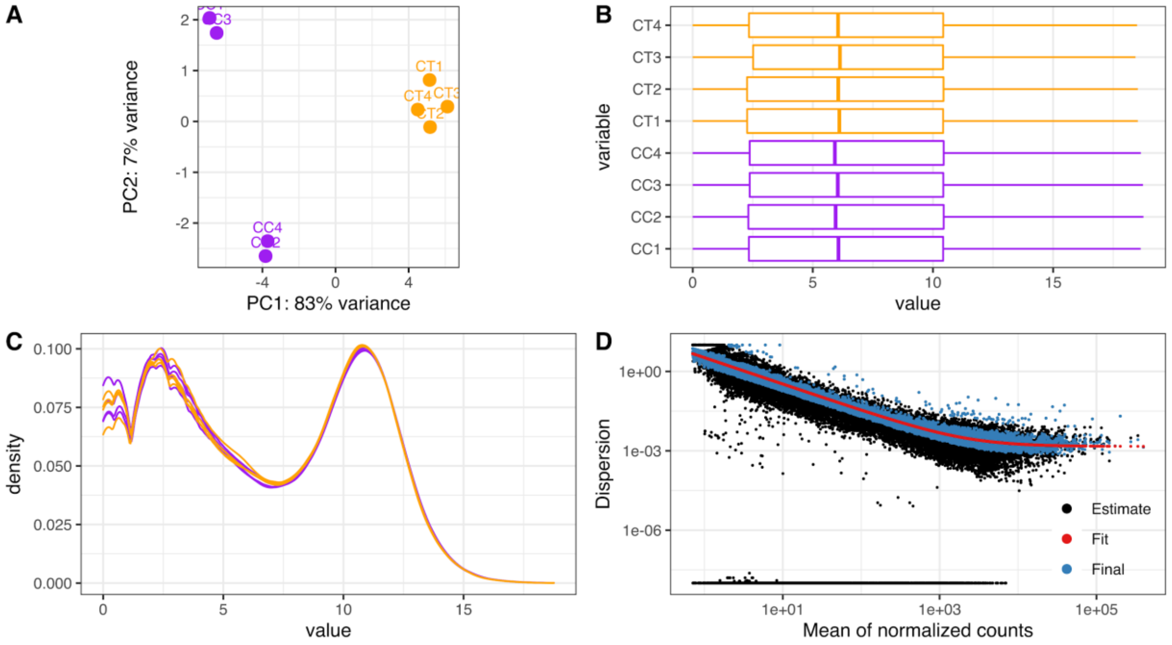


**Figure S1. Quality control for transcriptomic analysis. (A)** Principal component analysis (PCA). It is observed that 83% of the variation was contributed by the treatment, while the intragroup variation was mainly explained by the controls, representing 7% of the variation. The clustering pattern was the expected one and no potential outliers were observed for the samples. **(B)** Counting distributions. Normalized count distributions are displayed as a boxplot. **(C)** Counts density graphs. For B and C the normalized gene count represents the general levels of gene expression between samples under the assumption that most of them are not differentially expressed. Therefore, the spread of normalized counts per gene is expected to be similar for each sample. All samples presented a very similar gene expression profile, which makes them comparable for differential expression analysis. **(D)** Scatter plot and the mean of the normalized counts. The spread decreased as the mean of the normalized counts for each gene increased, indicating a good data set.

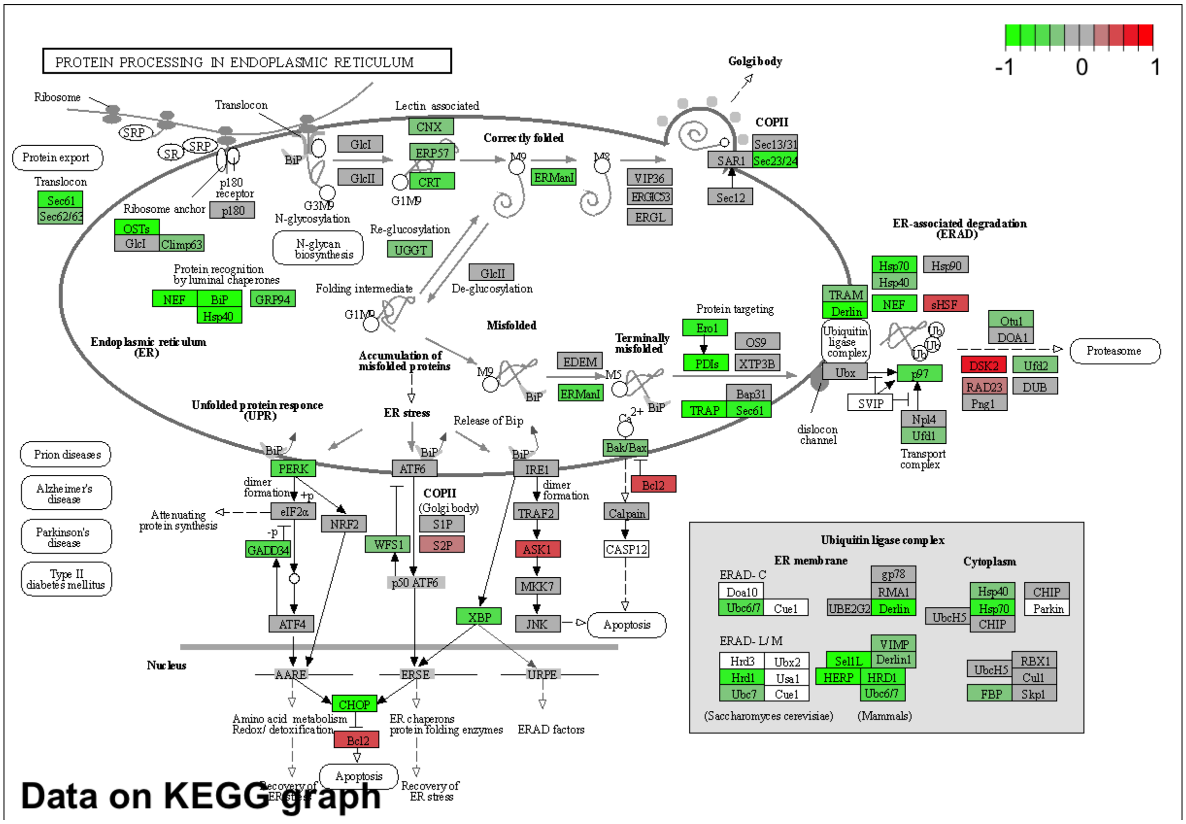


**Figure S2. Enriched pathway of KEGG (hsa04141) with FC for each gene.** DESeq2 log2 (FC) values for differentially expressed genes are mapped into the protein processing signaling pathway in the endoplasmic reticulum. (Red: up-regulated genes; green: down-regulated genes).
